# Classification of EEG Signals Using a Multiple Kernel Learning Support Vector Machine

**DOI:** 10.3390/s140712784

**Published:** 2014-07-17

**Authors:** Xiaoou Li, Xun Chen, Yuning Yan, Wenshi Wei, Z. Jane Wang

**Affiliations:** 1 Shanghai Medical Instrumentation College, Shanghai 200093, China; 2 School of Medical Instrument and Food Engineering, University of Shanghai for Science and Technology, Shanghai 200093, China; E-Mail: xiaouli@gmail.com; 3 Department of Biomedical Engineering, Hefei University of Technology, Hefei 230009, China; 4 Department of Neurology, Huadong Hospital Affiliated to Fudan University, Shanghai 200040, China; E-Mails: quack008@126.com (Y.Y.); wenshiwei@medmail.com.cn (W.W.); 5 Department of Electrical and Computer Engineering, University of British Columbia, Vancouver, BC V6T 1Z4, Canada; E-Mail: zjanew@ece.ubc.ca

**Keywords:** brain computer interface, mental task, stroke patients, multiple kernel learning, polynomial kernel, radial basis function kernel

## Abstract

In this study, a multiple kernel learning support vector machine algorithm is proposed for the identification of EEG signals including mental and cognitive tasks, which is a key component in EEG-based brain computer interface (BCI) systems. The presented BCI approach included three stages: (1) a pre-processing step was performed to improve the general signal quality of the EEG; (2) the features were chosen, including wavelet packet entropy and Granger causality, respectively; (3) a multiple kernel learning support vector machine (MKL-SVM) based on a gradient descent optimization algorithm was investigated to classify EEG signals, in which the kernel was defined as a linear combination of polynomial kernels and radial basis function kernels. Experimental results showed that the proposed method provided better classification performance compared with the SVM based on a single kernel. For mental tasks, the average accuracies for 2-class, 3-class, 4-class, and 5-class classifications were 99.20%, 81.25%, 76.76%, and 75.25% respectively. Comparing stroke patients with healthy controls using the proposed algorithm, we achieved the average classification accuracies of 89.24% and 80.33% for 0-back and 1-back tasks respectively. Our results indicate that the proposed approach is promising for implementing human-computer interaction (HCI), especially for mental task classification and identifying suitable brain impairment candidates.

## 1. Introduction

A brain computer interface (BCI) system is generally composed of a set of sensors and signal processing units, which can establish an information communication channel between a subject's brain and an external device. The realization of electroencephalography (EEG)-based BCI systems mainly involves three processes: first, the brain activity is recorded by means of electrodes located on the scalp and then a pre-processing step is applied to remove artifacts in order to enhance the signal-to-noise ratio; second, a feature extraction step is performed to extract meaningful information from raw EEG signals; the last step is conducted to translate such specific features into effective control commands and drive the external device. Since the implementation of BCI does not depend on peripheral nerves and muscles, it is particularly useful for individuals who suffer from the motor disorder with the cognitive ability [1–6].

EEG-based BCI technologies can be divided into many categories. In this study, we concentrate on the mental task recognition [7] and the P300 evoked related potential (ERP) recognition [8]. Keirn and Aunon [9] proposed that it was possible to accurately distinguish between various mental tasks using only the EEG. Many classification algorithms have been developed to improve mental task-based BCI performance due to the difficulty in obtaining high classification accuracy [10]. Garrett *et al.* [11] applied one linear (*i.e.*, linear discriminant analysis (LDA)) and two nonlinear (neural network (NN) and support vector machine (SVM)) classifiers to the classification of spontaneous EEG during five mental tasks. Palaniappan [10] used the spectral power differences in four bands and a NN classifier to classify different mental tasks. Li *et al.* [12] studied the classification of mental task EEG signals using SVM. Gupta *et al.* [13] used the relevant features with the SVM and LDA techniques to classify mental tasks.

Some studies have been also motivated by the goal of using EEG with effective algorithms to identify cognitive impairment patients. Lehmann *et al.* explored the ability of a multitude of linear and non-linear classification algorithms (*i.e.*, LDA, NN, SVM) to discriminate between EEG signals of patients with varying degrees of cognitive impairment [14]. Dauwels *et al.* used LDA and quadratic discriminant analysis (QDA) to classify cognitive impairment [15]. Gallego-Jutglà *et al.* used theta band power and LDA to achieve the best accuracy for diagnosing cognitive impairment [16].

In those EEG-based classification algorithms, although LDA has a very low computational requirement, it is not suitable for solving nonlinear problems [17]. NN can make the classification more flexible. However, the NN classifier requires a large number of training data and is also sensitive to over-fitting, especially for noisy and nonstationary data such as the EEG. This is because NN based on empirical risk minimization cannot control the learning mode well and requires more empirical parameters [18]. SVM based on structural risk minimization yields good performance in many applications, especially for solving problems with high dimension, nonlinearity and small dataset [19].

In SVM, a kernel function is an essential element, which maps samples in one feature space to another feature space. However, it is often unclear what the most suitable kernel for a task at hand is, and hence the user may wish to combine several possible kernels. One problem with simply adding kernels is that using equal weights is possibly not optimal. For instance, if one kernel is not correlated with the classification problem at all, then assigning it a great weight will degrade the performance. Multiple kernel learning SVM (MKL-SVM) is an efficient way of optimizing kernel weights [20,21]. Compared with a single kernel SVM, MKL-SVM can enhance the interpretability of the decision function and improve the performance [22–24].

For a BCI system, a major component is efficient feature extraction from multi-channel EEG signals. In recent years, as useful tools for analyzing the complexity and rhythm information of EEG signals, several popular approaches for the extraction of quantitative EEG features have been introduced, such as wavelet packet entropy (WPE), causality analysis, *etc.* Since EEG signals are non-stationary (both time-varying and space-varying), these methods are excellent candidates for the feature extraction process [25,26].

In this study, WPE was extracted from the mental task EEG. Granger causality flow was extracted from the cognitive task EEG. Then these features and the MKL-SVM classifier were used to perform the recognition of different tasks. The mental tasks were baseline task, visual counting task, mental letter composing task, mathematical multiplication task, and geometric figure rotation task, respectively. The recognition of 2-class, 3-class, 4-class, and 5-class cases of mental task EEG signals were performed. The cognitive tasks based on working memory that may elicit a P300 ERP component were 0-back and 1-back tasks. The recognition of stroke patients and healthy controls was also performed.

## 2. Classification Algorithm

The BCI performance mainly depends on the features and the classification algorithm adopted. The recognition efficiency of the classification algorithm is a key issue. Several popular algorithms have been developed to serve certain scenarios, such as LDA, NN and SVM [10–16]. These classical algorithms will be briefly reviewed. Comparatively, SVM is a powerful approach for pattern recognition especially for high dimensional, nonlinear problems. SVM has achieved better classification results in mental task and cognitive task classifications. Recent developments on SVM have shown the need to consider multiple kernels [23]. This provides flexibility and reflects the fact that typical learning problems often involve multiple, heterogeneous data sources. For appropriately designed kernels, the optimized combination weights can be used to understand which features are important for a specific recognition problem. In this study, we applied the MKL-SVM algorithm to the classifications of mental task and cognitive task EEG signals.

### 2.1. Brief Review of Popular Classification Algorithms

The LDA classifier is a dimension reduction method which finds an optimal linear transformation that maximizes the class separability. It performs well when the feature vector is multivariate normally distributed in each class group and different groups have a common covariance. However, these assumptions are rare in practice.

The NN classifier is a data driven self-adaptive method and provides a direct estimation of the posterior probabilities. It can be viewed as a mapping function, *F*: *R^d^* → *R^M^*, where *d*-dimensional input is submitted to the network and an M-vector network output is obtained to make the classification decision. Although the NN classifier can detect complex nonlinear relationships, its disadvantages include higher computational cost and the empirical nature of the model, *etc.*

The SVM algorithm is based on the statistical learning theory and the Vapnik-Chervonenkis dimension [27]. It maps the input patterns into a higher dimension feature space through some nonlinear mapping where a linear decision surface is then constructed [28]. In SVM, a kernel function implicitly maps samples to a feature space given by a feature mapping [29]. Since there are different kernel types, it is often unclear what the most suitable kernel for a task at hand is, and hence the user may wish to combine several possible kernels. It is thus desirable to use an optimized kernel function that can fit the data structure well [21]. To optimally combine multiple kernels in SVM, the MKL-SVM approach is introduced as follows.

### 2.2. Multiple Kernel Learning SVM

We employed an algorithm called SimpleMKL that Rakotomamonjy *et al.* [30] proposed to solve the MKL-SVM problem. The kernel of SimpleMKL is defined as a linear combination of multiple kernels. The algorithm is essentially based on a gradient descent on the SVM objective value through a weighted 2-norm regularization formulation with an additional constraint on the weights. It can be applied to multiclass classification beyond binary classification, meanwhile it has rapid convergence and favourable efficiency compared with other algorithms [31–33].

SVM is developed from the optimal hyperplane in the case of linear separating. In MKL-SVM, any dataset (*x_i_*, *y_i_*), where *x_i_* ∈ ℝ*^n^*, *y_i_* ∈ {−1,1}, can be separated by an optimal separating hyperplane: Σ*m f_m_* (*x*)+*b*=0 with the maximum margin between two classes. The constrained optimization problem for MKL-SVM is described as [30]:

(1) $$\begin{array}{ll} & \underset{d}{\min}J(d)\overset{M}{\underset{m=1}{\text{∑}}}d_{m}=1,d_{m}\geq 0,\forall m\\ J\left(d\right)= & \{\begin{array}{c} \underset{\left\{f_{m}\right\},b,\xi ,d}{\min}\frac{1}{2}\underset{m}{\text{∑}}\frac{1}{d_{m}}{\left\|f_{m}\right\|}_{{ℋ}_{m}}^{2}+C\underset{i}{\text{∑}}\xi_{i}\forall i\\ s.t.y_{i}\underset{m}{\text{∑}}f_{m}(x_{i})+y_{i}b\geq 1-\xi_{i}\\ \xi_{i}\geq 0\forall i \end{array} \end{array}$$

where ξ*_i_* is a loose variable. ξ*_i_* = 0 represents a linear separable case, ξ*_i_* > 0 represents a linear nonseparable case with certain misclassification. The penalty factor *C* controls the degree of misclassification. *M* is the total number of kernels used. *d_m_* is the weight coefficient of the kernel function. Each basic kernel *K_m_* corresponds to one *f_m_*. Each *d_m_* controls the squared norm of *f_m_* in the objective function. The smaller *d_m_* is, the smoother *f_m_* will be. Equation (1) can be transformed into the following dual form using Lagrange multipliers:

(2) $$J\left(d\right)=\{\begin{array}{c} \underset{\alpha }{\max}-\frac{1}{2}\underset{i,j}{\text{∑}}\alpha_{i}\alpha_{j}y_{i}y_{j}\underset{m}{\text{∑}}d_{m}K_{m}(x_{i},x_{j})+\underset{i}{\text{∑}}\alpha_{i}\forall i\\ s.t.\underset{i}{\text{∑}}\alpha_{i}y_{i}=0\\ C\geq \alpha_{i}\geq 0\forall i \end{array}.$$

This is a typical dual problem. Only a small number of α*_i_* are nonzero, and they correspond to some data points that are support vectors.

For the nonlinear case, the original problem can be solved by mapping the original data space into a high dimension feature space 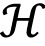 with a mapping Ø: ℝ*^n^* → 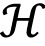. It is unnecessary to exactly know the mapping Ø(*x*) if we use the kernel function *K*(*x_i_*, *x_j_*) = (Ø(*x_i_*)·Ø(*x_j_*)), which is a symmetric function and satisfies the Mercer condition [21]. The classical kernel functions include polynomial function (Poly) *K_poly_* (*x*, *y*) = ((*x*·*y*)+1)*^d^*, radial basis function (RBF) *K_RBF_* (*x*,*y*) = exp (−‖*x*− *y2/2δ2*) and sigmoidal function (Sig) *KSigx,y* =tanh (*kx*·*y*+*v*), *etc*.

*d_m_* is updated with the descent direction. The updating scheme is *d* ← *d* + *γD*, where *γ* is the step size. This procedure is repeated until the objective value stops decreasing. The descent direction *D* is defined as:

(3) $$D_{m}=\{\begin{array}{l} 0d_{m}=0,\frac{\partial J}{\partial d_{m}}-\frac{\partial J}{\partial d_{\mu }}>0\\ -\frac{\partial J}{\partial d_{m}}+\frac{\partial J}{\partial d_{\mu }}d_{m}>0,m\neq \mu \\ \underset{v\neq \mu ,d_{v}>0}{\text{∑}}\left(\frac{\partial J}{\partial d_{v}}-\frac{\partial J}{\partial d_{\mu }}\right)m=\mu \end{array}$$

where $\frac{\partial J}{\partial d_{m}}=-\frac{1}{2}\sum_{i,j}\alpha_{i}\alpha_{j}y_{i}y_{j}K_{m}(x_{i},x_{j})$.*d_μ_* is the maximal nonzero component of *d*. *d_v_* is a component corresponding to the maximal admissible step size.

The algorithm is terminated at the following duality gap [30]:

(4) $$\underset{m}{\max}\sum_{i,j}\alpha_{i}\alpha_{j}y_{i}y_{j}K_{m}\left(x_{i},x_{j}\right)-\sum_{i,j}\alpha_{i}\alpha_{j}y_{i}y_{j}\sum_{m}d_{m}K_{m}\left(x_{i},x_{j}\right)\leq \varepsilon$$

The optimal classification function is:

(5) $$\{\begin{array}{l} f\left(x\right)=\text{sgn}(\underset{sv}{\text{∑}}\alpha_{i}y_{i}K(x,x_{i})+b)\\ K\left(x,x_{i}\right)=\overset{M}{\underset{m=1}{\text{∑}}}d_{m}K_{m}(x,x_{i}) \end{array}$$

## 3. Methods

The presented approach consisted of three main parts: (1) the purpose of the pre-processing step was to improve the general signal quality of the EEG in order to get more accurate rhythm analysis and measurements; (2) the features including WPE and Granger causality were extracted from the EEG signals to compose a feature vector for further classification; (3) MKL-SVM was employed to perform the different task classification, and the classification accuracies were used to evaluate the performance of the proposed algorithm.

### 3.1. Human Subjects

#### 3.1.1. EEG Data of Mental Task

The EEG data used in this study were collected by Keirn and Aunon [9]. Seven subjects, 21 to 48 years old, participated in the experiment. An elastic electrode cap was used to record EEG signals. The electrodes were placed on the scalp at C3, C4, P3, P4, O1, and O2 based on the international 10–20 system. They were referenced to two electrically linked mastoids, A1 and A2. The data were sampled at 250 Hz. Signals were recorded for 10 s during each task. Each task was repeated for two sessions. Each session contained five trials. The data are available online at http://www.cs.colostate.edu/∼anderson. Five different mental tasks were involved [10], namely:

*Baseline Task (denoted by task B)*: The subjects were told to relax and try to think of nothing.
*Visual Counting Task (denoted by task C)*: The subjects were told to imagine a blackboard and visualise numbers being written on the board sequentially, with the previous number being erased before the next number was written.
*Mental Letter Composing Task (denoted by task L)*: The subjects were told to mentally compose a letter to a friend or a relative without vocalising.
*Mathematical Multiplication Task (denoted by task M)*: The subjects were given nontrivial multiplication problems, e.g., 24 times 14 and were told to solve them without vocalising or making any other physical movements.
*Geometric Figure Rotation Task (denoted by task R)*: The subjects were given 30 s to study a particular three-dimensional block object, after which the drawing was removed and the subjects were told to visualise the object being rotated about an axis.

#### 3.1.2. EEG Data of Cognitive Task

Consecutive patients aged 50 years or older with a first-ever acute ischemic stroke at Huadong Hospital Affiliated to Fudan University were recruited. All patients underwent cognitive testing, and those who met the criteria for mild cognitive impairment were included (*n* = 13). 13 age- and sex-matched healthy controls were enrolled in this cross-sectional study. All subjects were right handed and had normal vision. This study was approved by Huadong Hospital Affiliated to Fudan University Ethics Board, and all subjects gave written, informed consents before participation.

**Figure 1.**
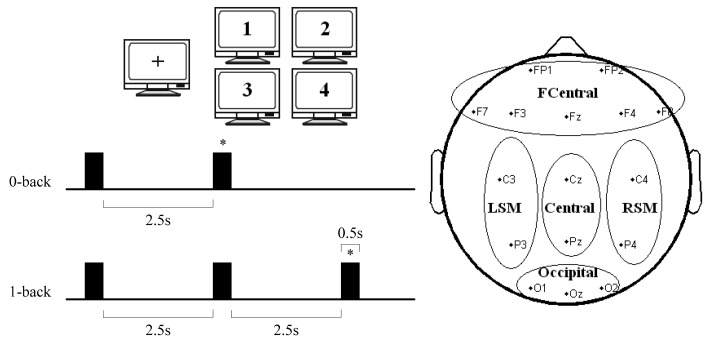
N-back task timeline and electrode positions. Here trial time sequences for 0-back and 1-back conditions. Black squares represent each stimulus in the task. The symbol * stands for the target number during each trial. Five brain regions: Fronto-Central (FCentral)-FP1, FP2, F7, F3, Fz, F4 and F8, Left Sensorimotor (LSM)-C3 and P3, Central- Cz and Pz, Right Sensorimotor (RSM) - C4 and P4, and Occipital-O1 and O2.

As shown in Figure 1, the working memory was assessed using a verbal N-back task [34]. A pseudorandom set of 4-digit numbers was displayed on a monitor, and the subjects were instructed to determine whether a specific digit–one appeared on the screen (0-back task); or the currently displayed number at any given time had been already displayed in the previous presentation (1-back task). Stimuli consisted in a 0.5 s. Inter stimulus interval (ISI) was 2.5 s in all conditions. Subjects had to distinguish between targets and non-targets by pressing a keyboard. Continuous ERP signals were acquired using an EEG/ERP amplifier system (NATION^®^ Inc., Shanghai, China). For all ERP recordings, 18 electrodes were placed according to the 10–20 international system. The chosen electrode positions were EOG1, EOG2, Fp1, Fp2, F3, F4, F7, F8, Fz, C3, Cz, C4, P3, Pz, P4, O1, Oz and O2 (Figure 1). The data were sampled at 256 Hz. Signals were recorded for 120 s during each task. Each task was repeated for three sessions. Each session contained 40 trials with a 1:1 target/non-target relation. Namely, the total number of targets was 60, the same as that of non-targets.

### 3.2. Recognition of Mental Task EEG

**Figure 2.**
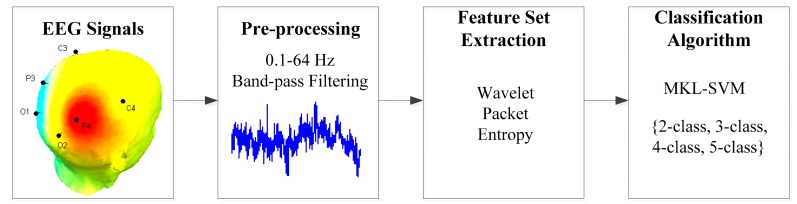
The basic diagram of mental task recognition.

We proposed a mental task-based BCI approach, as illustrated in Figure 2.

WPE can characterize the physiological state changes of the subjects during different mental tasks [12]. It provides an effective way for studying the complexity of EEG signals that can decode specific function states from the brain activity, and the obtained model from the time sequence and the estimated parameters can reveal the mechanism of EEG.

Brain electrical activity mapping (BEAM) summarizes the EEG data as color maps. Li *et al.* [12] studied the BEAM at 2.9 Hz, 6.1 Hz, 10 Hz, and 22 Hz of five different mental states and found that different mental tasks had different energy distributions in the brain area. They correspond to specific frequency bands in the EEG, such as 0–4 Hz (delta), 4–8 Hz (theta), 8–12 Hz (alpha), and 12–32 Hz (beta). The wavelet packet transform (WPT) can be viewed as a generalization of the classical wavelet transform, which provides a multi-resolution and time-frequency analysis for nonstationary EEG signals with a binary tree structure. The entropy provides a measurement of the signal uncertainty.

As mentioned above, we extracted four frequency band data (*i.e.*, delta, theta, alpha, and beta) of mental task EEG signals through the WPT decomposition, then calculated their entropies to analyze locally nonlinear features that can reflect the complexity distribution of the signal in each frequency band. A wavelet packet is represented as a function [25]:

(6) $$\psi_{j,k}^{i}\left(t\right)=2^{-\frac{j}{2}}\psi^{i}\left(2^{-j}t-k\right),i=1,2,\cdots ,j^{n}$$

where *i* is the modulation parameter, *j* is the dilation parameter, *k* is the translation parameter, *n* is the level of the decomposition in the wavelet packet tree, and ψ*^i^* is called as a mother wavelet.

The wavelet packet coefficients $c_{j,k}^{i}$ corresponding to the signal *f*(*t*) can be obtained as:

(7) $$c_{j,k}^{i}(t)=\int_{-\infty }^{\infty }f(t)\psi_{j,k}^{i}(t)dt$$

The wavelet packet component of the signal at a particular node can be obtained as:

(8) $$f_{j}^{i}(t)=\overset{\infty }{\underset{k=-\infty }{\text{∑}}}c_{j,k}^{i}\psi_{j,k}^{i}\left(t\right)dt$$

The Shannon entropy $SE_{f_{j}^{i}}$ can be defined as the entropy of the signal component $f_{j}^{i}$, $SE_{f_{j}^{i}}$is written as:

(9) $$SE_{f_{j}^{i}}=-\sum_{i}{f_{j}^{i}}^{2}\log({f_{j}^{i}}^{2})$$

### 3.3. Recognition of Cognitive Task P300

**Figure 3.**
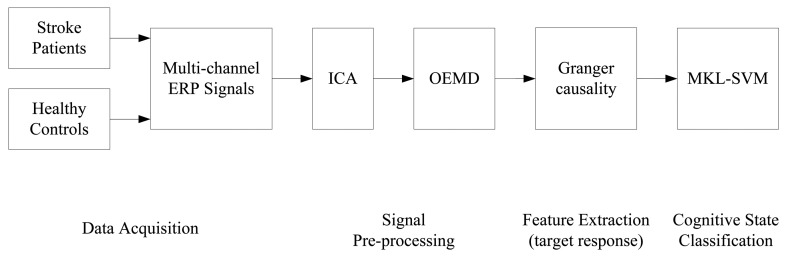
The general diagram of cognitive task recognition.

We proposed a classification approach of stroke patients and healthy controls, as illustrated in Figure 3.

#### 3.3.1. Pre-Processing

Independent component analysis (ICA) is a kind of blind source separation technique that extracts statistically independent sources called independent components (ICs) from a set of recorded signals. Orthogonal empirical model decomposition (OEMD) is a self-adaptive signal processing and data driven method. Compared with classical time-frequency analysis methods, such as short time Fourier transform (STFT) and wavelet, it is based on the local characteristic time scales of a signal and could decompose the signal into a set of complete orthogonal components called intrinsic mode functions (IMFs) which are determined by the signal itself without prior knowledge about the signal [35,36].

In this study, ICA and OEMD were combined to extract more informative features, which can be used as inputs to a classifier to improve the classification accuracy of task-related activity. We first extracted statistically independent sources from the given ERP signals by ICA, and then decomposed them into spectrally independent modes using OEMD algorithm.

#### 3.3.2. Feature Extraction

Understanding and modeling the brain function is based not only on the correct identification of the active brain regions, but also on the functional interactions among the neural assemblies distributed across different brain regions. Correlation, coherence, Granger causality and information entropy have been widely used for the estimation of effective connectivity from the EEG data in the space-frequency and space-time-frequency domains. In particular, Granger causality is one of the prototypical data-driven effective connectivity techniques. It can describe the direct or indirect information flow that one neural system exerts another neural system and quantify causal interactions between brain sources [26,37]. According to the principle of Granger causality, *X*_2_ causes *X*_1_ (*F*_2→1_) if the inclusion of past observations of *X*_2_ reduces the prediction error of *X*_1_ in a linear regression model of *X*_1_ and *X*_2_, as compared to a model which includes only previous observations of *X*_1_. Two time series *X*_1_(*t*) and *X*_2_(*t*) can be defined as [38]:

(10) $$\begin{array}{c} X_{1}\left(t\right)=\overset{p}{\underset{j=1}{\text{∑}}}A_{11,j}X_{1}\left(t-j\right)+\overset{p}{\underset{j=1}{\text{∑}}}A_{12,j}X_{2}\left(t-j\right)+e_{1}\left(t\right)\\ X_{2}(t)=\overset{p}{\underset{j=1}{\text{∑}}}A_{21,j}X_{1}\left(t-j\right)+\overset{p}{\underset{j=1}{\text{∑}}}A_{22,j}X_{2}\left(t-j\right)+e_{2}(t) \end{array}$$

where *p* is the maximum number of lagged observations included in the autoregressive (AR) model, *A* represents the AR coefficients, *e*_1_(*t*) and *e*_2_(*t*) are the residuals.

The magnitude of *F*_2→1_ in the time domain can be obtained by the log ratio of the prediction error variances, as follows:

(11) $$F_{2\to 1}=ln\frac{\text{var}(e_{1R(12)})}{\text{var}(e_{1U})}$$

where *e*_1_*_R_*_(12)_ is derived from the AR model omitting the *A*_12,_*_j_* coefficients and *e*_1_*_U_* is derived from the full AR model.

The spectral Granger causality from *X*_2_ to *X*_1_ can be defined as:

(12) $$I_{2\to 1}\left(f\right)=-\text{ln}\;\left(1-\frac{({\text{∑}}_{22}\text{−}(\sum_{12}^{2}/\sum_{11})){\left|H_{12}(f)\right|}^{2}}{S_{11}(f)}\right)$$

where ∑ is the noise covariance matrix, *H* is the transfer matrix and S is the power spectrum of *X*_1_ at frequency *f* [38].

**Figure 4.**
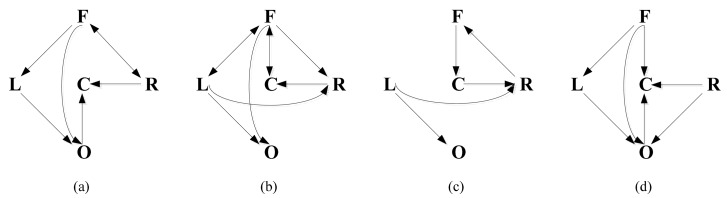
Brain connectivity patterns of stroke patients and healthy controls. (**a**) Stroke patients in 0-back task; (**b**) Healthy controls in 0-back task; (**c**) Stroke patients in 1-back task. (**d**) Healthy controls in 1-back task.

In this study, we analyzed the spectral Granger causality in the theta band. To simplify the analysis, the EEG signals were divided into five regions. They were FCentral (F), LSM (L), Central (C), RSM (R) and Occipital (O), respectively. The raw signals of the electrodes within each region were averaged as the overall activity of the region. Figure 4 shows the average causal network between brain sources for stroke patients and healthy controls in 0-back and 1-back tasks. The connectivity pattern difference between stroke patient group and healthy control group was obvious (*t* = 3.8621, *p* < 0.05).Compared with healthy controls, stroke patients showed a connectivity reduction, especially for the left cortex. There were some functional disconnections among different cortical regions for stroke patients, such as F↔C and L→R in 0-back task, F→L, O→C and F→O in 1-back task. From an anatomical standpoint, the cognitive disturbances associated with the information transmission may not solely be due to the loss of neurons, but also due to the impairment of distributed neuronal activity [37–40].

### 3.4. Parameter Settings

In this study, all EEG signals were classified via a 5-fold cross-validation. 80% of all samples were used for training and 20% for testing.

When WPE was used, the EEG signals in each channel were decomposed into 4 levels through the WPT decomposition with the db4 wavelet. We used D40, D41, D42 and (D43, D21) node to reconstruct the signal, which corresponded to delta, theta, alpha and beta frequency bands respectively. WPE feature was computed for each frequency band.

For the proposed MKL-SVM algorithm, three Poly kernels and ten RBF kernels were chosen. The Poly kernel is a global kernel which has a good generalization ability and a low learning capacity, while the RBF kernel is a local kernel which has a poor generalization ability and a high learning capacity. The overall performance of the kernel function may be improved by their linear combination. In the Poly kernel, if the freedom degree is too high, the generalization ability will decrease, and the over-fitting problem may occur. The degrees (*d*) of Poly kernels were chosen as [1, 2, 3]. In the RBF kernel, the small Gaussian width is available for severe changeable samples. The large Gaussian width is available for mild changeable samples. The widths (*δ*) of RBF kernels were chosen as [0.5, 1, 2, 5, 7, 10, 12, 15, 17, 20]. The kernel parameters chosen in the following single kernel SVM also came from these parameters.

SVM was originally designed for binary classification, but can be extended to the multiclass classification cases. Several approaches have been suggested for multiclass classification using SVM, and here we adopted the one-versus-rest approach. In this approach, a set of binary classifiers, each of which is trained to separate one class from the rest, are undertaken and each test sample is allocated to the class for which the largest decision value is determined [21].

## 4. Results

### 4.1. Kernel Weights

**Figure 5.**
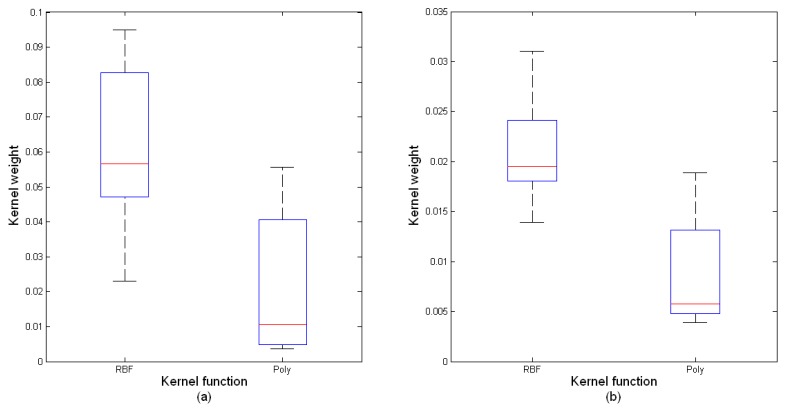
Boxplots of kernel weights for mental task and cognitive task classifications. (**a**) Mental task classification; (**b**) Cognitive task classification.

The MKL-SVM algorithm combines several possible kernels through optimizing kernel weights. The weight values vary with different classification tasks. Figure 5 shows the boxplots of kernel weights for mental task and cognitive task classifications. In general, the weight values of RBF kernels were greater than the ones of Poly kernels and more RBF kernels were favorable.

### 4.2. Mental Task Classification

The mental task combinations used for the classification are shown in Table 1. Corresponding to the WPE feature, the accuracies of 2-class, 3-class, 4-class, and 5-class classification are presented in Table 2. The average accuracies over all seven subjects were 99.20%, 81.25%, 76.76%, and 75.25% respectively.

**Figure 6.**
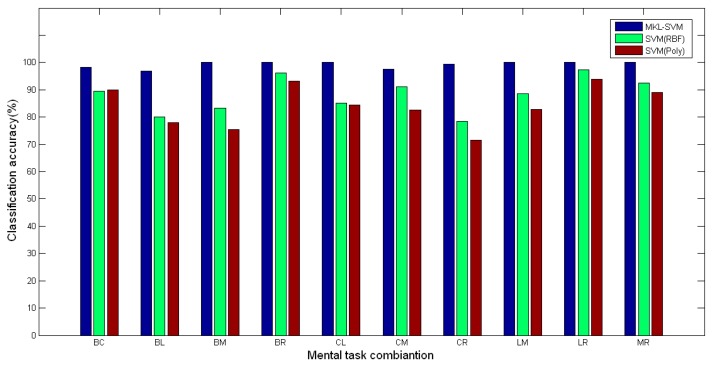
Accuracy comparisons of 2-class classification cases. In the legend, the notation “SVM (RBF)” represents the SVM algorithm with the RBF kernel. The notation “SVM (Poly)” represents the SVM algorithm with the Poly kernel.

For 2-class classification, all subjects can completely recognize BM, BR, CL, LM, LR and MR pair tasks. Their classification accuracies were all 100%. The classification accuracy of the BL classification was the lowest, 96.94%. Therefore, it was easy for all subjects to recognize mathematical multiplication and geometric figure rotation tasks, with respect to the baseline state. In addition, the combination of mathematical multiplication and geometric figure rotation tasks was also easy to be distinguished. This supports the neuroscience knowledge that the brain can make fine distinction between calculation and visual tasks, because the left hemisphere of the brain is dominant for the calculation task, while the right hemisphere of the brain is dominant for the visual task [41]. The accuracy of 2-class classification using MKL-SVM was further compared with the single kernel SVM when using same features. The comparison results are shown in Figure 6. As can be seen, the classification results based on MKL-SVM were better than those based on SVM with the Poly kernel (*t* = 6.306, *p* < 0.05) and the RBF kernel (*t* = 5.308, *p* < 0.05). Besides, RBF had better results than Poly. These results indicated that the overall classification accuracy of MKL-SVM was much higher than the accuracy of the SVM algorithm based on a single kernel.

In all ten 3-class mental task classification cases, the average accuracy for BMR classification was the highest, 89.93%, while CLM was the lowest, 74.31%.

In all five 4-class mental task classifications, the average accuracy for BLMR classification was the highest, 83.14%. The average accuracy for BCLM classification was the lowest, 71.09%.

The sensitivity degrees for certain mental tasks in subjects varied, and there were individual differences for the adaptability for the mental task experiment. We noted that the most discriminatory tasks differed among the subjects, which was consistent with the reported studies [42]. None of the subjects had the perfect classification performance for CLM and BCLM. It could be noted from the results that most of the subjects may well recognize BMR tasks due to the high difference in the baseline, mathematical multiplication and geometric figure rotation tasks. From a physiological point of view, the region and level of the excitation in the cerebral cortex are different during different mental tasks. Compared with other regions, the EEG generated from the active area of the cortex is obvious.

**Figure 7.**
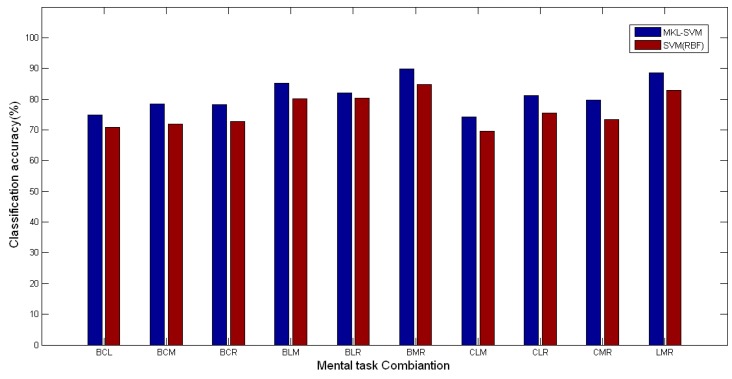
Accuracy comparisons of 3-class classification cases.

**Figure 8.**
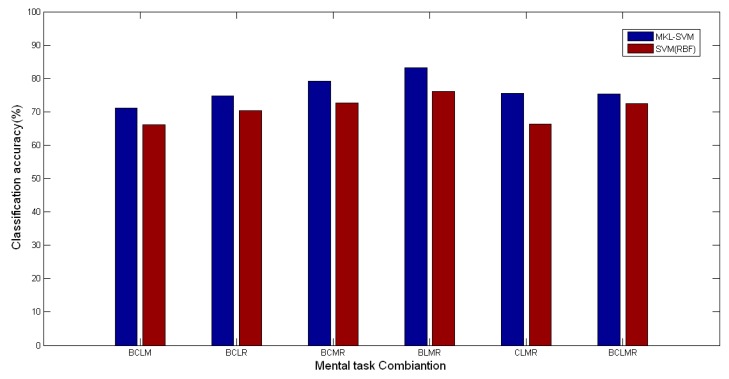
Accuracy comparisons of 4-class and 5-class classification cases.

Zhang *et al.* [43] studied the multiclass classification from four subjects using the frequency band powers and asymmetry ratios from the frequency range of 0 to 100Hz with the Fisher discriminant analysis, in which the average accuracies for 3-class, 4-class, and 5-class classifications were 65.88%, 58.18%, and 52.75% respectively (different sizes of training set and test set). Comparatively, the performance of the proposed algorithm was better. As mentioned above, SVM with the RBF kernel performed better than that with the Poly kernel. We also compared the average accuracy using MKL-SVM with SVM based on single RBF kernel. The comparisons of 3-class classification are shown in Figure 7 (*t* = 2.108, *p* < 0.05). The comparisons of 4-class and 5-class classifications are shown in Figure 8 (*t* = 2.719, *p* < 0.05). We note that MKL-SVM performed consistently better. From Figures 7 and 8, we also noted that the same best mental task combinations were observed for both MKL-SVM and the single kernel SVM, *i.e.*, BMR and BLMR. This observation suggested that the most easily recognized tasks were rather independent of the classification methods in most cases.

### 4.3. Cognitive Task Classification

Table 3 shows the classification comparison results of 0-back and 1-back tasks distinguishing stroke patients *versus* healthy controls between MKL-SVM and another two single kernel SVM algorithms with the spectral causality flow feature. The average classification accuracy for 0-back task was 89.24%. The average classification accuracy for 1-back task was 80.33%. As has been shown, the classification results based on MKL-SVM were better than those based on Poly kernel (*t* = 6.221, *p* < 0.05) and RBF kernel (*t* = 4.187, *p* < 0.05). The general classification accuracy of 0-back task was higher than that of 1-back task. These results provide theoretical and experimental basis of the quantity diagnosis for cognitive impairment. It is helpful for the intelligent identification of cognitive function and appropriate rehabilitation training.

### 4.4. Computational Cost

The proposed MKL-SVM algorithm was implemented in MATLAB 7.12.0 on a personal computer (using an Intel(R) Core(TM) i5-2430M CPU@2.40 GHz, 2.91 GB RAM). The average runtime varied from 6.86 s to 25.78 s. It included the cross validation and the optimization process. The average runtime with SVM was from 5.44 s to 20.47 s.

## 5. Conclusions

In this paper, we have presented the MKL-SVM algorithm for the classifications of mental tasks and working memory tasks with WPE and Granger causality features. The proposed method yielded better performance than the single kernel SVM. It can achieve 99.20% average accuracy for 2-class classification and above 75% for multiclass classification. It achieved above 89% accuracy for 0-back task and above 80% for 1-back task. The average CPU runtime was 15.39 s for the training and testing. Therefore, it can be a practical method for EEG-based BCI. Meanwhile, the classification results showed that the most discriminatory tasks varied among different subjects. In general, BMR and BLMR were the most suitable mental tasks to be distinguished. The Granger causality results showed that the effective connections among different cortical regions were reduced in stroke patients.

The physiological phenomena whereby different types of mental activities can activate distinct areas of the cortex and evoke different EEG rhythms made it possible to classify mental tasks using EEG signals and to realize a BCI system based on the conversion of different mental tasks. For example, invalids can express their demands to the carers and people can operate home computers using only their mind through the BCI. It is also an effective method to implement working memory task-based BCI based on cognitive impairment. First, we can perform the accurate identification and assessment of cognitive function by the EEG signal detection. Then, the rehabilitation training for cognitive impairment may be employed by the BCI with the appropriate apparatus.

## Figures and Tables

**Table 1.** The classification problems for different mental task combinations. The five mental tasks are represented by B, C, L, M and R, as described in Section 3.1.1. Here the notation “BC” is used to represent the 2-class classification problem of mental tasks B and C (baseline, visual counting). The notation “BCL” is used to represent the 3-class classification problem of mental tasks B, C and L (baseline, visual counting and letter composing). The notation “BCLM” is used to represent the 4-class classification problem of mental tasks B, C, L and M (baseline, visual counting, letter composing and multiplication). The other notations are similarly defined.

2-Class Classification	3-Class Classification	4-Class/5-Class Classification
BC	BCL	BCLM
BL	BCM	BCLR
BM	BCR	BCMR
BR	BLM	BLMR
CL	BLR	CLMR
CM	BMR	BCLMR
CR	CLM	
LM	CLR	
LR	CMR	
MR	LMR	

**Table 2.** Classification accuracies for mental tasks over all seven subjects. Here S1 represents the first subject. The other notations are similarly defined.

Subject	Average Classification Accuracies (%)			

	2-Class	3-Class	4-Class	5-Class
S1	98.24	73.95	73.14	66.15
S2	100	83.78	78.46	72.22
S3	100	80.89	69.62	75.00
S4	100	84.89	88.17	85.71
S5	96.61	79.72	78.64	67.64
S6	99.58	87.17	70.99	85.04
S7	100	78.33	78.33	75.00
Mean	99.20	81.25	76.76	75.25

**Table 3.** Classification accuracies for 0-back and 1-back tasks with different classifiers (%). Here SP represents stroke patient, HC represents healthy control.

Classification Tasks	Classifiers	Classification Accuracies (%)
0-back (SP-HC)	MKL-SVM	89.24
	SVM(RBF)	81.61
	SVM(Poly)	77.14

1-back (SP-HC)	MKL-SVM	80.33
	SVM(RBF)	75.28
	SVM(Poly)	70.81
